# Mosquito Hemocytes Associate With Circulatory Structures That Support Intracardiac Retrograde Hemolymph Flow

**DOI:** 10.3389/fphys.2018.01187

**Published:** 2018-08-28

**Authors:** Leah T. Sigle, Julián F. Hillyer

**Affiliations:** Department of Biological Sciences, Vanderbilt University, Nashville, TN, United States

**Keywords:** immunity, circulation, phagocytosis, heart, dorsal vessel, *Anopheles gambiae*

## Abstract

A powerful immune system protects mosquitoes from pathogens and influences their ability to transmit disease. The mosquito's immune and circulatory systems are functionally integrated, whereby intense immune processes occur in areas of high hemolymph flow. The primary circulatory organ of mosquitoes is the dorsal vessel, which consists of a thoracic aorta and an abdominal heart. In adults of the African malaria mosquito, *Anopheles gambiae*, the heart periodically alternates contraction direction, resulting in intracardiac hemolymph flowing toward the head (anterograde) and toward the posterior of the abdomen (retrograde). During anterograde contractions, hemolymph enters the dorsal vessel through ostia located in abdominal segments 2–7, and exits through an excurrent opening located in the head. During retrograde contractions, hemolymph enters the dorsal vessel through ostia located at the thoraco-abdominal junction, and exits through posterior excurrent openings located in the eighth abdominal segment. The ostia in abdominal segments 2 to 7—which function in anterograde intracardiac flow—are sites of intense immune activity, as a subset of hemocytes, called periostial hemocytes, respond to infection by aggregating, phagocytosing, and killing pathogens. Here, we assessed whether hemocytes are present and active at two sites important for retrograde intracardiac hemolymph flow: the thoraco-abdominal ostia and the posterior excurrent openings of the heart. We detected sessile hemocytes around both of these structures, and these hemocytes readily engage in phagocytosis. However, they are few in number and a bacterial infection does not induce the aggregation of additional hemocytes at these locations. Finally, we describe the process of hemocyte attachment and detachment to regions of the dorsal vessel involved in intracardiac retrograde flow.

## Introduction

The dorsal vessel of an insect is the main pulsatile organ that drives hemolymph circulation throughout the hemocoel (Jones, 1977; Chapman et al., 2013; Klowden, 2013; Wirkner et al., 2013; Hillyer, 2015). It is a contractile tube that traverses the length of the body and is comprised of the aorta in the head and thorax, and the heart in the abdomen. Hemolymph enters the dorsal vessel through valves called ostia and is propelled by the wave-like contractions of heart muscle. Depending on the insect or life stage, the dorsal vessel propels hemolymph toward the head (anterograde) or periodically alternates between propelling hemolymph toward the head and toward the posterior of the body (retrograde).

**Figure 1:**
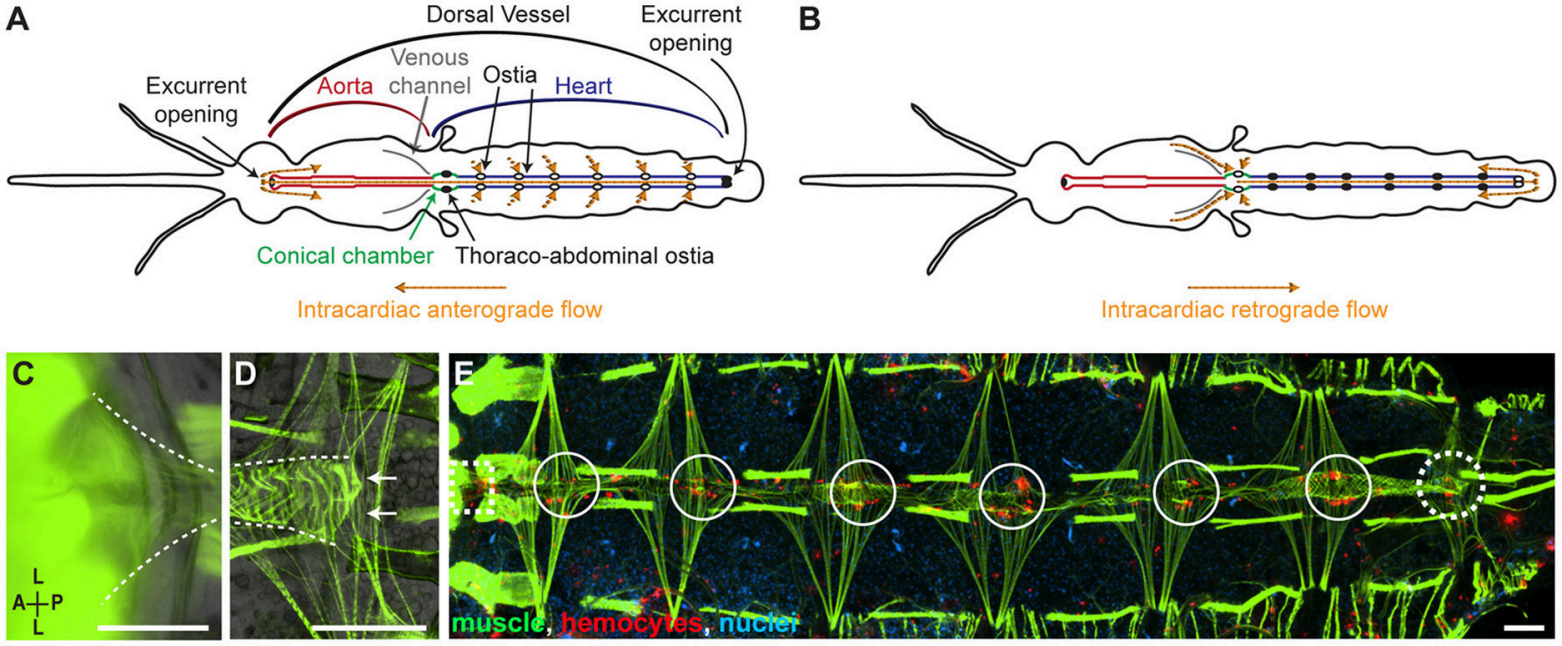
The hemocytes of naïve mosquitoes are present at sites of incurrent and excurrent intracardiac retrograde hemolymph flow. **(A,B)** Dorsal view of the structure of the circulatory system of an adult mosquito showing the flow of hemolymph during anterograde **(A)** and retrograde **(B)** heart contractions. Diagrams are adapted from Sigle and Hillyer, 2018b. **(C,D)** Dissected specimens showing the muscles (phalloidin; green) of the conical chamber at the thoraco-abdominal junction **(C**; dotted lines) and the posterior excurrent openings (arrows) of the heart (**D**; dotted lines). **(E)** Dissected naïve mosquito where muscle (green) and hemocytes (CM-DiI; red) have been fluorescently labeled, showing the presence of hemocytes around the thoraco-abdominal ostia (dotted square), the periostial regions of abdominal segments 2–7 (solid circles), and the posterior excurrent openings of the heart (dotted circle). Scale bars: 100 μm.

The heart of the adult malaria mosquito *Anopheles gambiae* alternates between contracting in the anterograde and retrograde directions (Figures 1A,B; Glenn et al., 2010). When the heart contracts anterograde, hemolymph enters the lumen of the dorsal vessel through 6 pairs of incurrent ostia located in the anterior portion of abdominal segments 2–7, and exits the vessel through an excurrent opening located in the head (Glenn et al., 2010). When the heart contracts retrograde, hemolymph in the venous channels of the thorax and in the hemocoel of the first abdominal segment enters the dorsal vessel through a pair of thoraco-abdominal ostia, and exits the vessel through a pair of excurrent openings located in the 8th abdominal segment (Glenn et al., 2010; Sigle and Hillyer, 2018b). The thoraco-abdominal ostia are in a region of the heart called the conical chamber, which is adjacent to the location where the heart, aorta, and venous channels converge. Though the heart is the primary circulatory pump, the aorta persistently contracts in the anterograde direction (Sigle and Hillyer, 2018b). However, hemolymph does not flow through the aorta during periods of retrograde heart contractions.

The circulatory and immune systems of insects are functionally integrated, and this integration has been described in detail in *A. gambiae* (Hillyer, 2015; League and Hillyer, 2016). In adult mosquitoes, a population of sessile hemocytes—called periostial hemocytes—are always present in the regions surrounding the abdominal ostia (the periostial regions), where they phagocytose pathogens in regions of high hemolymph flow (King and Hillyer, 2012; Sigle and Hillyer, 2016). Upon infection, additional hemocytes actively migrate to the periostial regions, where they aggregate and continue the phagocytosis and killing of pathogens. The aorta lacks ostia, and hence, even though a few hemocytes are distributed across its surface, infection does not induce the aggregation of hemocytes in that portion of the dorsal vessel (Sigle and Hillyer, 2018b).

Whereas the presence and aggregation of hemocytes at the periostial regions of abdominal segments 2–7 has been clearly established, it is unclear whether hemocytes are present at the thoraco-abdominal ostia. This distinction is important because the abdominal ostia function in anterograde heart flow whereas the thoraco-abdominal ostia function in retrograde heart flow (Glenn et al., 2010; Sigle and Hillyer, 2018b). Furthermore, whereas few or no hemocytes are present in the anterior excurrent opening of the aorta—a structure that functions during anterograde heart flow—it remains unknown whether hemocytes are present at the posterior excurrent openings of the heart—a structure that functions during retrograde heart flow (Glenn et al., 2010; Sigle and Hillyer, 2018b). In this study we examined hemocyte presence and function at sites of the dorsal vessel that are important during periods of intracardiac retrograde hemolymph flow. We uncovered immunologically active hemocytes around the thoraco-abdominal ostia and the posterior excurrent openings of the heart. However, these hemocytes are few in number, and infection does not induce their aggregation at these locations. Finally, by means of intravital video imaging we revealed that hemocyte associations with circulatory structures involved in intracardiac retrograde flow are dynamic in that hemocytes attach to and detach from these structures.

## Materials and methods

### Mosquito rearing and maintenance

*Anopheles gambiae* Giles sensu stricto (G3 strain) were reared and maintained at 27°C and 75% relative humidity under a 12 h: 12 h light: dark photoperiod as previously described (Estévez-Lao et al., 2013). Larvae were fed a mixture of koi food and yeast, and adults were fed 10% sucrose solution *ad libitum*. Experiments were initiated on adult female mosquitoes at 5 days post-eclosion, an age when infection is known to induce the aggregation of hemocytes at the periostial regions of the heart (King and Hillyer, 2012; Sigle and Hillyer, 2016, 2018a).

### Mosquito injections and bacterial infection

For all injections, mosquitoes were anesthetized on ice and then injected into the hemocoel a volume of 0.15–0.20 μl using a glass needle that had been inserted through the thoracic anepisternal cleft as previously described (Coggins et al., 2012). For infections, tetracycline resistant/GFP-expressing *Escherichia coli* (DH5 alpha) were grown in Luria-Bertani media (LB) and injected. The infection dose was determined immediately after each experiment by plating serial dilutions of the bacterial cultures (Coggins et al., 2012), and averaged 72,560 *E. coli* per mosquito. To control for the effect of injection, a subset of mosquitoes was injected sterile LB (injury group).

### Mosquito dissections

Mosquitoes were anesthetized on ice, injected with 16% formaldehyde to fix hemocytes and other tissues (Electron Microscopy Sciences, Hatfield, PA, USA), and the head, legs and wings were removed by cutting with a fine blade. To isolate the dorsal thoraco-abdominal junction and abdomen, a mosquito was placed in PBS containing 0.1% Tween-20 (Fisher Scientific, Pittsburgh, PA, USA), the abdomen and thorax were bisected along the coronal plane, and the thorax was bisected at the anterior-posterior midline of the transverse plane. The internal organs were removed to expose the dorsal vessel, and the specimen was placed in Aqua Poly/Mount (Polysciences, Warrington, PA, USA) on a microscope slide. To resect the heart, the heart was pulled away from the dorsal cuticle after disrupting all alary muscles with an insect pin.

### Fluorescence labeling

To label muscle, mosquitoes were anesthetized, injected with 16% formaldehyde, and incubated for 5 min. Mosquitoes were dissected to expose the relevant structures and then incubated in a solution of 0.6 μM phalloidin-Alexa Fluor 488 (to label muscle green; Invitrogen, Carlsbad, CA, USA), 0.75 mM Hoechst 33342 (to label nuclei blue; Invitrogen) and 0.1% Triton X-100 (Fisher Scientific) in PBS for 30 min. Afterwards, specimens were washed 3 times in PBS.

The hemocytes of live mosquitoes were fluorescently labeled red with CM-DiI, which is a dye that when injected into the hemocoel becomes incorporated in hemocytes and no other cells (King and Hillyer, 2012). Briefly, mosquitoes were anesthetized, injected a solution of 75 mM Vybrant CM-DiI Cell-Labeling Solution (Invitrogen) and 0.75 mM Hoechst 33342 in PBS, and incubated at 27°C for 20 min. Mosquitoes were then either imaged intravitally through the dorsal cuticle, further processed to label muscle, or dissected to either visualize structures or count hemocytes. In specimens where muscle and hemocytes were both labeled, following CM-DiI labeling, muscle was stained as described above or by intrathoracic injection of phalloidin-Alexa Fluor 488 as previously described (Glenn et al., 2010), with the exception that Triton X-100 was not included in the solution (Sigle and Hillyer, 2018b).

Hemocytes were also labeled by means of their phagocytosis of *E. coli* bacterial bioparticles conjugated to pHrodo (Sigle and Hillyer, 2016). For these experiments, *E. coli*-pHrodo-Red bioparticles (Invitrogen) were reconstituted in PBS at 1 mg/ml, injected into live mosquitoes, and mosquitoes were maintained at 27°C for 24 h. Mosquitoes were then anesthetized, injected with 16% formaldehyde, and either imaged through the dorsal cuticle or were dissected and washed 3 times in PBS. These specimens were either mounted for imaging or further processed for muscle staining.

### Acquisition of still images

Images were acquired on a Nikon 90i compound microscope (Nikon Corp, Tokyo, Japan) equipped with a linear encoded Z-motor, a Nikon Intensilight C-HGFI fluorescence illumination unit, a Nikon DS-Qi1Mc CCD camera, and Nikon Advanced Research NIS-Elements software. Three-dimensional Z-stack images were acquired and rendered into focused two-dimensional images using the Extended Depth of Focus tool in NIS-Elements.

### Counting of hemocytes

CM-DiI-labeled hemocytes were observed and counted at 24 h post-treatment in naïve, injured (LB injected) and *E. coli* infected mosquitoes. At the thoraco-abdominal junction, hemocytes were visualized through the dorsal cuticle of intact mosquitoes. This approach was taken because disruption of the thoracic flight muscles during dissection causes the non-specific incorporation of CM-DiI into the myofibers, and this interferes with hemocyte visualization in the thorax and the first abdominal segment. This technique was validated by comparing hemocyte counts in intact mosquitoes and the same mosquitoes after dissection, which revealed that more hemocytes could be identified in the first abdominal segment of intact mosquitoes. In the 8th abdominal segment, hemocyte counts were similar regardless of whether specimens were visualized before or after dissection, so hemocytes were counted in intact specimens.

Following CM-DiI labeling and incubation at 27°C, mosquitoes were anesthetized on ice, injected with 16% formaldehyde, and the head, legs, and wings were removed by cutting with a fine blade. Specimens were placed dorsal-side-up for visualization under epi-fluorescence illumination at 200–400X magnification and the number of hemocytes at the thoraco-abdominal ostia and the posterior excurrent openings were counted. For a cell to be considered a hemocyte it had to be 9–18 μm in diameter (King and Hillyer, 2012; Hillyer and Strand, 2014; Sigle and Hillyer, 2016), and it had to be labeled with both CM-DiI and Hoechst 33342. A minimum of 25 mosquitoes across 7 independent trials were analyzed for each treatment group, and for each mosquito, data were collected for both the thoraco-abdominal ostia and the posterior excurrent openings. Data were analyzed with the non-parametric Kruskal-Wallis test and Spearman correlation analysis using Prism 6 Software (GraphPad, La Jolla, CA, USA).

### Intravital video imaging

Live mosquitoes were visualized in real-time through the dorsal abdominal cuticle. Hemocytes were labeled with CM-DiI, and mosquitoes were restrained dorsal-side-up using a non-invasive method previously described (Boppana and Hillyer, 2014). Mosquitoes were imaged on the Nikon 90i compound microscope, and real-time videos were acquired under low-level epi-fluorescence illumination (using an ND 2 filter) at a magnification of 100X.

## Results

### Hemocytes attach to the regions surrounding the thoraco-abdominal ostia and the posterior excurrent openings

When the heart contracts in the retrograde direction, hemolymph enters the lumen of the dorsal vessel through a single pair of ostia located at the thoraco-abdominal junction, and exits the heart via the posterior excurrent openings (Figures 1A–D). To determine whether hemocytes associate with structures involved in intracardiac retrograde hemolymph flow, we examined whether—similar to what occurs in the periostial regions of abdominal segments 2 through 7—hemocytes are present at the thoraco-abdominal junction. In addition, we examined whether hemocytes are present at the excurrent openings of the 8th abdominal segment. In naïve mosquitoes, hemocytes labeled with CM-DiI were often observed in the areas surrounding the thoraco-abdominal ostia as well as near the posterior excurrent openings of the heart (Figure 1E). However, not all mosquitoes had hemocytes at these locations. At the thoraco-abdominal ostia and the excurrent openings, hemocytes were present in 79 and 46% of mosquitoes, respectively, and presence at the thoraco-abdominal ostia was not always a predictor of presence at the posterior excurrent openings (and vice versa; Spearman's correlation *p* = 0.850). Together, these findings show that hemocytes often surround circulatory structures involved in intracardiac retrograde hemolymph flow.

### Infection does not increase the number of hemocytes on the thoraco-abdominal ostia and the posterior excurrent openings

**Figure 2:**
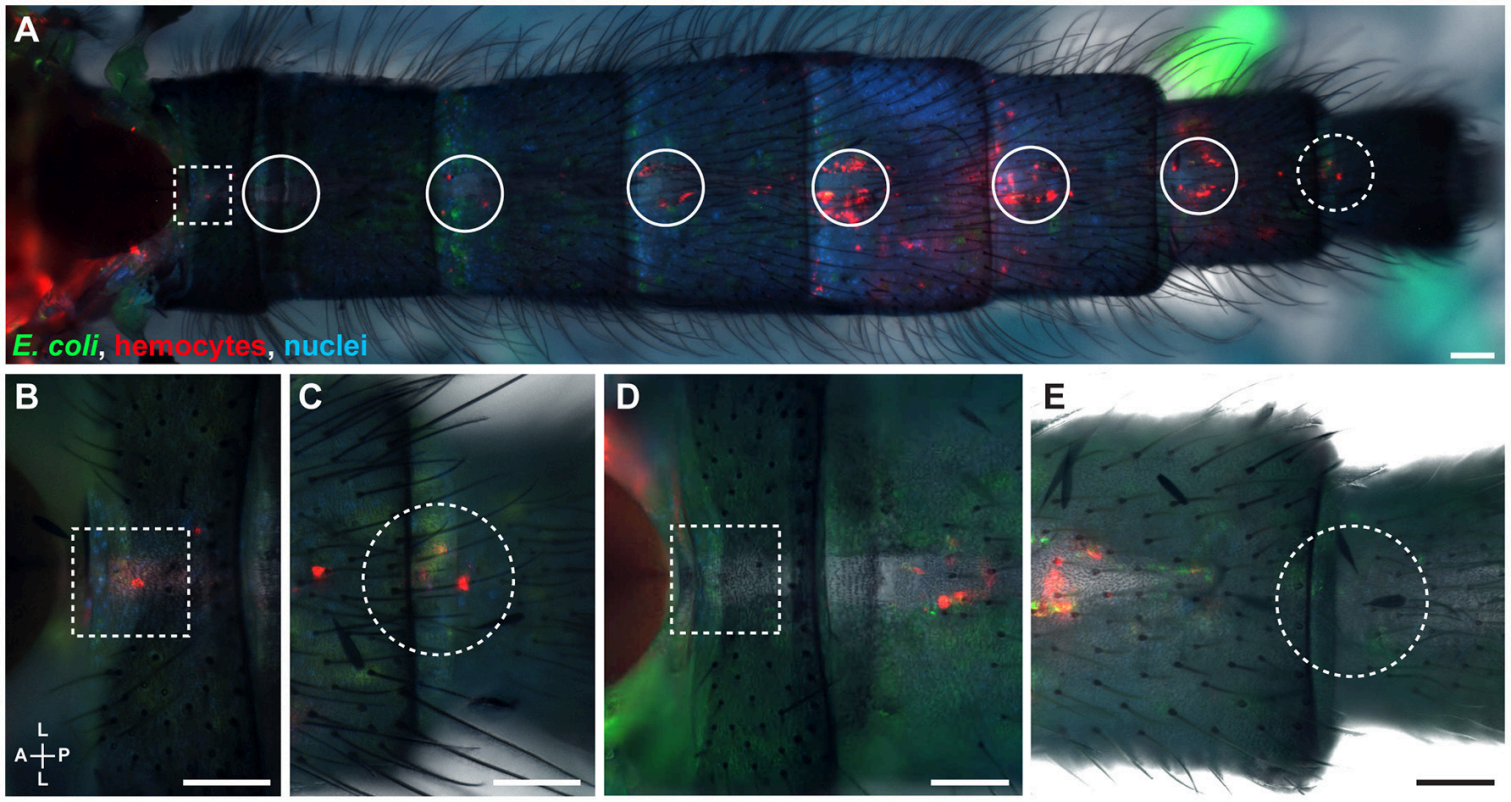
The hemocytes of infected mosquitoes are present at sites of incurrent and excurrent intracardiac retrograde hemolymph flow. **(A–C)** Intact infected mosquito showing hemocytes (red) and *E. coli*-GFP (green) around the thoraco-abdominal ostia **(A**, magnified in **B**; dotted squares) and the posterior excurrent openings of the heart (**A**, magnified in **C**; dotted circles). **(D,E)** Images of another infected mosquito showing that hemocytes do not always surround the thoraco-abdominal ostia **(D**; dotted square) or the posterior excurrent openings **(E**; dotted circle). Nuclei were labeled blue with Hoechst 33342. Scale bars: 100 μm.

**Figure 3:**
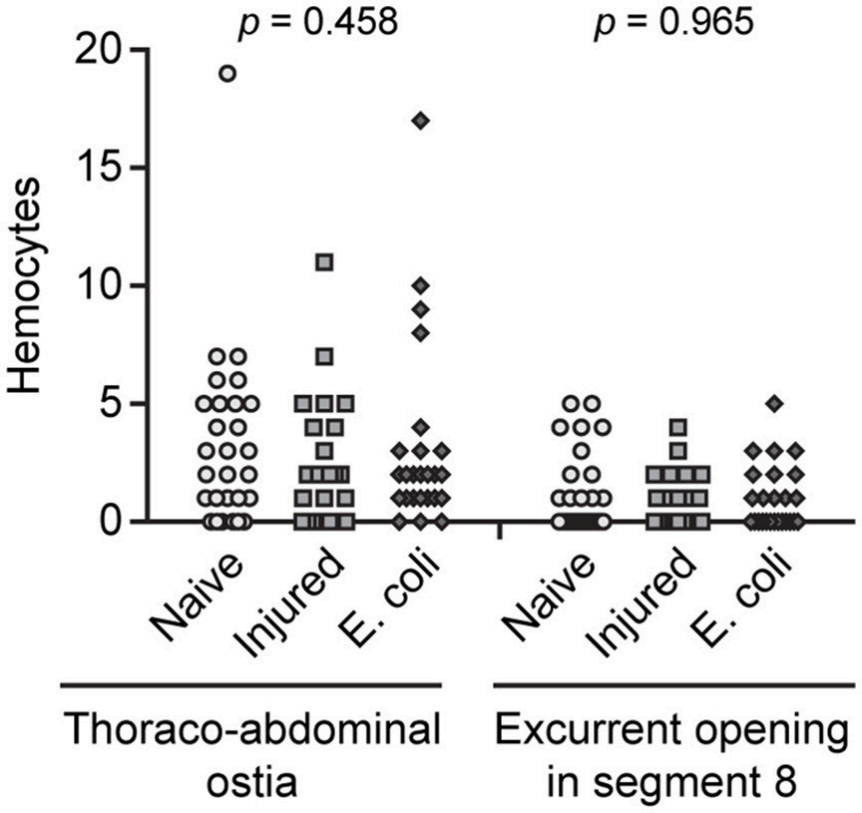
Hemocytes are few in number at sites of incurrent and excurrent intracardiac retrograde hemolymph flow and do not increase in response to infection. Number of hemocytes at the thoraco-abdominal ostia and the posterior excurrent openings of the heart of naïve, injured and *E. coli* infected adult mosquitoes at 24 h following treatment. Each point represents the number of hemocytes in an individual mosquito, and *p*-values result from a Kruskal-Wallis test.

**Figure 4:**
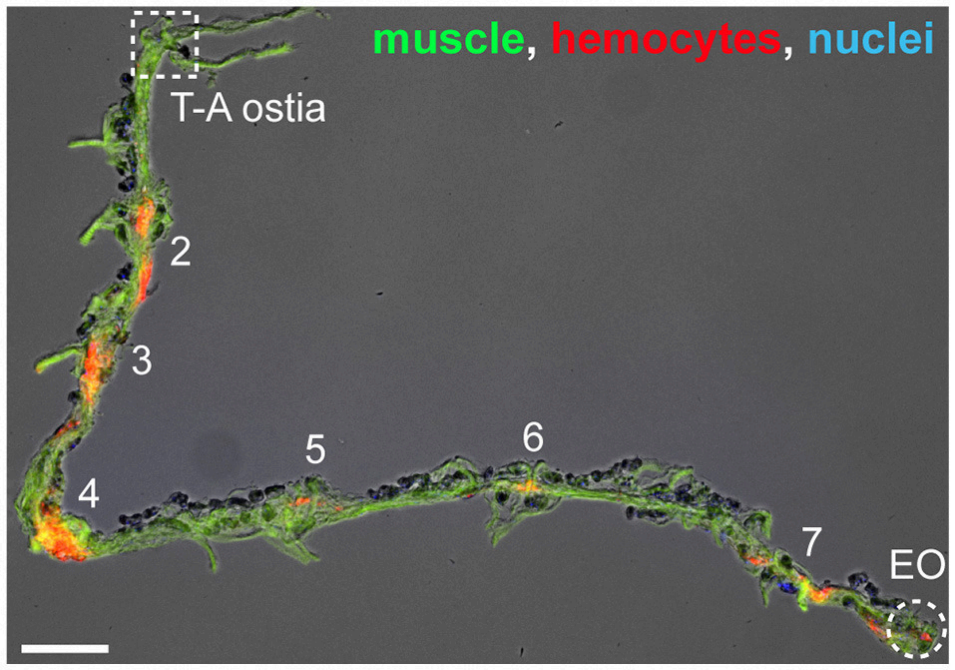
Hemocytes on a resected heart. Image of a resected heart where muscle (phalloidin; green), hemocytes (CM-DiI; red), and nuclei (Hoechst 33342; blue) have been labeled. In this specimen there are no hemocytes at the thoraco-abdominal ostia (T-A ostia), but there are hemocytes at the periostial regions (abdominal segments are numbered) and at the posterior excurrent openings (EO). Scale bar: 100 μm.

Because infection induces the aggregation of hemocytes at the periostial regions (King and Hillyer, 2012; Sigle and Hillyer, 2016), we hypothesized that infection increases the number of hemocytes at the thoraco-abdominal ostia, and perhaps the posterior excurrent openings. To test this hypothesis, we visualized and counted the hemocytes present at these locations in naïve, injured, and *E. coli* infected mosquitoes at 24 h post-treatment. Similar to naïve mosquitoes, some infected mosquitoes had hemocytes at these structures whereas others did not (Figures 2A–E). Specifically, mosquitoes had a median of 2.5 and 2.0 hemocytes at the thoraco-abdominal ostia of naïve and injured mosquitoes, respectively (Figures 3). At 24 h following *E. coli* infection, the median number of hemocytes was also 2.0, indicating that infection does not cause hemocyte aggregation at this location (Kruskal-Wallis *p* = 0.458). A similar trend was observed at the posterior excurrent openings. Naïve, injured and *E. coli*-infected mosquitoes had a median number of 0, 0.5, and 0 hemocytes at the posterior excurrent openings, respectively (Figures 3; Kruskal-Wallis *p* = 0.965), and never more than 5. There was also no correlation between the number of hemocytes at the thoraco-abdominal ostia and the number of hemocytes at the posterior excurrent openings (Spearman *r* = −0.189, 0.180, and 0.338 for naïve, injured and infected mosquitoes, respectively; Spearman's correlation *p* = 0.336, 0.378, and 0.098 for naïve, injured and infected mosquitoes, respectively). Even though few hemocytes were present at the structures involved in intracardiac retrograde hemolymph flow, examination of intact, dissected or resected specimens consistently confirmed the presence of numerous periostial hemocytes in abdominal segments 2–7, and that their number increases following infection (Figures 1E, 2A, 4).

### Hemocytes at the thoraco-abdominal ostia and the posterior excurrent openings are phagocytic

**Figure 5:**
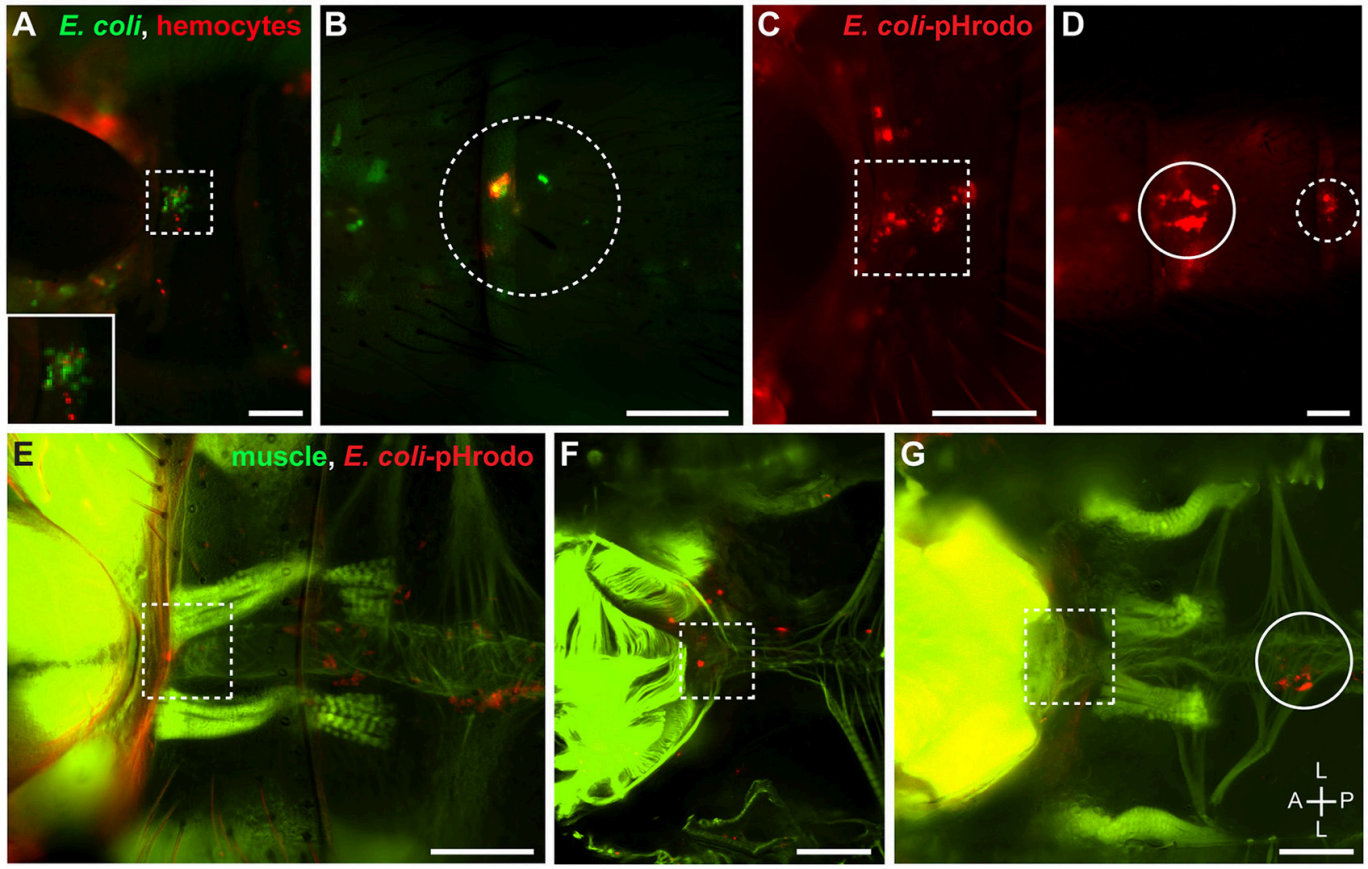
Hemocytes at the thoraco-abdominal ostia and the posterior excurrent openings are phagocytic. **(A,B)** Intact adult mosquito showing that *E. coli*-GFP (green) co-localizes with hemocytes (CM-DiI; red) that surround the thoraco-abdominal ostia **(A**; dotted square; inset) and the posterior excurrent openings of the heart **(B**; dotted circle). **(C,D)** Intact adult mosquito showing that hemocytes that surround the thoraco-abdominal ostia **(C)** and the posterior excurrent openings of the heart **(D)** phagocytose *E. coli*-pHrodo (red). **(E,F)** Adult mosquitoes showing phagocytic hemocytes (*E. coli*-pHrodo) on the muscle (phalloidin; green) surrounding the thoraco-abdominal ostia in intact **(E)** and dissected **(F)** specimens. **(G)** Dissected adult mosquito showing that phagocytic hemocytes are not always present at the thoraco-abdominal ostia. In panels **(D,G)**, phagocytic hemocytes can be seen in the periostial regions of the 7th and 2nd abdominal segments, respectively (solid circles). Scale bars: 100 μm.

To determine whether the hemocytes present at the thoraco-abdominal junction and the posterior excurrent openings are immunologically active, we tested their phagocytic activity. After infection, hemocytes at the thoraco-abdominal ostia and the posterior excurrent openings co-localized with *E. coli*-GFP, suggesting that pathogens are actively phagocytosed and killed by hemocytes at these locations (Figures 5A,B). To confirm phagocytosis, mosquitoes were injected pHrodo-conjugated *E. coli* bacterial bioparticles. These dead bacteria are conjugated to a pH-sensitive dye that only fluoresces in acidic environments, such as the phagolysosome. As expected, many hemocytes—including those present at the thoraco-abdominal ostia, the posterior excurrent openings, and the periostial regions—readily phagocytosed *E. coli*-pHrodo (Figures 5C–G).

### Hemocyte interactions with structures involved in intracardiac retrograde flow are dynamic

During retrograde heart contractions, hemolymph flowing through the venous channels of the thorax or the dorsal hemocoel of the first abdominal segment enters the conical chamber of the heart via the thoraco-abdominal ostia Figure 1B; Sigle and Hillyer, 2018b). Intravital imaging of the thoraco-abdominal ostia revealed that most hemocytes enter the conical chamber by first flowing through the venous channels of the thorax, and a smaller proportion of hemocytes enters the heart by first flowing in the hemocoel of the first abdominal segment (Supplementary Video 1). Hemocytes move through the venous channels at high speeds, which may hinder their ability to attach to the thoraco-abdominal ostia. Imaging of the 8th abdominal segment revealed that there is significant movement of hemocytes at the posterior excurrent openings of the heart. Hemocytes exiting the heart swiftly flow through the excurrent openings, and sessile hemocytes on the posterior surface of the heart are periodically released back into circulation (Supplementary Video 2). Thus, these data show the dynamic movement of hemocytes at locations of intracardiac retrograde hemolymph flow, and their association and dissociation with circulatory structures.

## Discussion

In this study we show that hemocytes surround structures that support intracardiac retrograde hemolymph flow. Though not always present, a small number of hemocytes are often located at the thoraco-abdominal ostia and the posterior excurrent openings of the heart. Infection does not induce the aggregation of hemocytes at these locations; however, the hemocytes present at the thoraco-abdominal ostia and the posterior excurrent openings are phagocytic and immunologically active.

Prior studies showed that mosquito hemocytes aggregate near circulatory structures involved in intracardiac anterograde flow (King and Hillyer, 2012; Sigle and Hillyer, 2016). Specifically, a population of hemocytes, called periostial hemocytes, surround the ostia of abdominal segments 2–7, where they phagocytose pathogens in regions of high hemolymph flow. Because these locations are only functional when the heart contracts anterograde (Glenn et al., 2010), we hypothesized that hemocytes are also present in circulatory structures involved in intracardiac retrograde hemolymph flow, such as the thoraco-abdominal ostia. We found that hemocytes are present at the thoraco-abdominal ostia of most but not all mosquitoes, but that they are few in number. The average naïve mosquito has two hemocytes at this location, which is fewer than the fifty or so hemocytes present at the periostial regions (King and Hillyer, 2012; Sigle and Hillyer, 2016). Furthermore, infection does not recruit additional hemocytes to the thoraco-abdominal ostia, which is different from the more than doubling of hemocytes that occurs at the periostial regions following infection. Thus, although all hemocytes, regardless of location, are immunologically active, the thoraco-abdominal ostia are not locations of intense immune activity, and infection-induced hemocyte aggregation is restricted to the abdominal periostial regions. Given that the heart of 5-day-old adult *A. gambiae* spends a significant amount of time (~1/3) contracting retrograde (Glenn et al., 2010; Estévez-Lao et al., 2013), and that the proportion of retrograde contractions increases with age (Doran et al., 2017), the lack of concerted immune activity at the thoraco-abdominal ostia was unexpected. We speculate that the absence of infection-induced hemocyte aggregation at the thoraco-abdominal ostia is due to the shear force of flow at this region, relative to the periostial regions of the abdomen. Another possible explanation is that hemocyte aggregation at this location would restrict flow at the only entry-point for hemolymph during retrograde heart contractions. Hemocytes assume an asymmetric distribution across the 6 periostial regions of the abdomen, with most hemocytes aggregating in segments 4–6. This asymmetric distribution is less pronounced in infected mosquitoes, suggesting that periostial hemocyte aggregation results in the partial obstruction of flow at the ostia of the mid-abdominal segments, which results in the partial redirection of hemolymph to other ostial pairs (Sigle and Hillyer, 2016). Perhaps obstructing hemolymph flow at the thoraco-abdominal ostia is costlier than obstructing flow at any of the 6 pairs of abdominal ostia.

On the surface, it appears that our finding that few hemocytes are present at the thoraco-abdominal ostia is in contrast to findings made in *Drosophila melanogaster*, where hemocytes and pathogens are aggregated—in high numbers—at the conical chamber (Elrod-Erickson et al., 2000; Horn et al., 2014; Ghosh et al., 2015). However, there are two clear distinctions between mosquitoes and fruit flies. The first distinction is that the conical chamber of mosquitoes has only one ostial pair—the thoraco-abdominal ostia—whereas the conical chamber of fruit flies has two ostial pairs—the thoraco-abdominal ostia and the first abdominal ostial pair (Wasserthal, 2007; Glenn et al., 2010; Sigle and Hillyer, 2018b). That posteriormost ostial pair of the conical chamber of fruit flies is functionally similar to the ostial pair of the second abdominal segment of mosquitoes—which is a location where periostial hemocytes aggregate. The second distinction is that a region surrounding the conical chamber of fruit flies serves as a hematopoietic hub (Ghosh et al., 2015). Such a hub has not been described in mosquitoes. Instead, mosquito hemocytes have been found to divide while in circulation, although it is possible that they replicate at a yet to be described sessile location (Christensen et al., 1989; Castillo et al., 2011; King and Hillyer, 2013; Bryant and Michel, 2014).

We also found that hemocytes are present at the posterior openings of the heart, but again, they are few in number and they do not aggregate at this location in response to infection. This is in contrast to what occurs in mosquito larvae (League and Hillyer, 2016), and some lepidopteran larvae (Locke, 1997), where numerous hemocytes exist attached to the tracheal tufts that surround the posterior of the heart. At least in mosquitoes, the reason for this difference pertains to changes in circulatory physiology that occur during development (League et al., 2015). That is, the adult heart contracts bidirectionally and the posterior openings have excurrent function whereas the larval heart only contracts anterograde and the posterior openings have incurrent function. In that sense, the posterior of the larval heart is functionally analogous to the abdominal ostia of adults. Thus, our findings at the posterior of the adult heart were not surprising, especially because hemocytes do not aggregate at the anterior end of the mosquito aorta (Sigle and Hillyer, 2018b), and experiments in fruit flies have not detected the aggregation of hemocytes or pathogens on the posterior of the heart (Horn et al., 2014; Ghosh et al., 2015).

Intravital video imaging of hemocytes revealed that their movement near the posterior excurrent openings is dynamic. Hemocytes can bind and detach from this location, indicating that hemocytes can readily change from circulating to sessile states and vice versa. We have previously detected similar attachment and detachment of hemocytes in the periostial regions of adult mosquitoes (Sigle and Hillyer, 2016). Furthermore, in addition to immunity, hemocytes are critical in development and wound healing (Krautz et al., 2014; Wood and Martin, 2017). In these instances, cells actively migrate to their sites of action (Wood et al., 2006; Babcock et al., 2008), and this migration is not visually dissimilar to the migration of mosquito hemocytes. Although the molecular basis of hemocyte migration has received significant attention in the *Drosophila* system (Evans and Wood, 2014), such information is unknown for mosquitoes. Significant strides have been made to uncover the transcriptome and proteome of mosquito hemocytes (Bartholomay et al., 2004; Baton et al., 2009; Pinto et al., 2009; Smith et al., 2016; Thomas et al., 2016; He et al., 2017; Severo et al., 2018), and we recently revealed that Nimrod family genes are involved in periostial hemocyte aggregation, but the relative roles of these genes—specifically *eater* and *draper*—in hemocyte migration requires further study (Sigle and Hillyer, 2018a). Further studies should seek to elucidate the relative contributions of the two major hemocyte populations—the phagocytic granulocytes and the melanizing oenocytoids (Hillyer and Strand, 2014)—on the immune responses that take place on the surface of the heart.

Mosquitoes transmit disease-causing pathogens. Many of these pathogens, such as *Plasmodium* sp., circulate with the hemolymph prior to invading their target organ: the salivary glands (Hillyer et al., 2007; Douglas et al., 2015). Hemocytes and hemocyte-derived factors attack these parasites while in the hemocoel, including at the periostial regions (Clayton et al., 2014; Hillyer and Strand, 2014; Severo and Levashina, 2014; Bartholomay and Michel, 2018). In this study we assessed hemocyte activity at two structures important for intracardiac retrograde hemolymph flow and found that infection does not induce the aggregation of hemocytes at the thoraco-abdominal ostia or the posterior excurrent openings of the heart. Together with data assessing immunity at the aorta and the periostial regions of the heart (King and Hillyer, 2012; Sigle and Hillyer, 2016, 2018b), these data show that the primary sites of immune activity on the dorsal vessel of adult mosquitoes are the incurrent structures involved in intracardiac anterograde hemolymph flow.

## Author contributions

LS and JH conceived and designed the study. LS conducted the experiments. LS and JH analyzed the data and wrote the manuscript.

### Conflict of interest statement

The authors declare that the research was conducted in the absence of any commercial or financial relationships that could be construed as a potential conflict of interest.
